# Investigation of the Mechanical Properties of Parts Fabricated with Ultrasonic Micro Injection Molding Process Using Polypropylene Recycled Material

**DOI:** 10.3390/polym12092033

**Published:** 2020-09-07

**Authors:** Rafael Gaxiola-Cockburn, Oscar Martínez-Romero, Alex Elías-Zúñiga, Daniel Olvera-Trejo, José Emiliano Reséndiz-Hernández, Cintya G. Soria-Hernández

**Affiliations:** Mechanical Engineering and Advanced Materials Department, School of Engineering and Science, Tecnologico de Monterrey, Ave. Eugenio Garza Sada 2501, Monterrey 64849, Mexico; A00960305@itesm.mx (R.G.-C.); daniel.olvera.trejo@tec.mx (D.O.-T.); emi_jose.18@tec.mx (J.E.R.-H.); cintya.soria@tec.mx (C.G.S.-H.)

**Keywords:** regrind, material recycling, granulation, ultrasonic micro injection molding, polypropylene, rheology

## Abstract

This research focuses on investigating how physical and mechanical properties of polypropylene (PP) recycled material are modified when ultrasonic micro injection molding (UMIM) technology is used to produce material specimens. Experimental characterization by differential scanning calorimetry (DSC), thermogravimetric analysis (TGA), Fourier transform infrared (FTIR) spectra, and rheology tests show that the fabricated PP samples were able to withstand up to five times recycled processing before some signs of mechanical and physical properties degradation are observed. Surprisingly, uniaxial extension tests show an increase of 3.07%, 10.97% and 27.33% for Young’s modulus, yield stress and ultimate stress values, respectively, and a slight reduction of 1.29% for the samples elongation at break when compared to the experimental data collected from virgin material samples. The improvement of these mechanical properties in the recycled samples suggests that ultrasonic microinjection produces a mechano-chemical material change.

## 1. Introduction

Up to now, only 17% of global plastics production is recycled annually [1]. Such a reduced percentage relies on the limitations and challenges that plastics impose, starting from its high handling cost, to the reduction of its mechanical and physical properties attained during recycling. Available data indicate that by 2015 the worldwide annual production of plastics reached more than 350 million tons. From this annual production, 171 million come from the industry of transportation, electric/electronics, packaging, automotive, aerospace, medical, and food industries, which are big consumers of injection molding parts [2]. Due to an increase in the demand for electrical and electronic components because of the Corporate Average Fuel Economy (CAFE) regulations, a yearly growth rate of almost 6% is expected in the injection molding (IM) industries for the year 2023 [3]. 

From the plastics processing methods, IM is one of the most common processes used to produce a variety of components, and evidently, the use of regrind (regranulated material) is a frequent task due to the residues generated in terms of sprues and runners that are solidified in every injection cycle, and post-consumer streams as well. The combination of reground resins for traditional IM has been studied. For instance, McLauchlin and Savage [4] studied the reprocessability of polyether ether ketone (PEEK). This study compared the effect of recycling against the properties of virgin material. Through tensile testing, X-Ray diffraction (XRD) and differential scanning calorimetry (DSC), they evaluated the mechanical properties, thermal behavior and crystallinity. They found that pure regrind PEEK can be reprocessed up to three cycles without presenting degradation in mechanical properties. Sobotova et al. [5] studied the impact of regrind in mechanical properties of polybutylene terephthalate (PBT) with 30% glass fiber. Tensile, Charpy impact, and hardness tests were performed by adding 20%, 40%, 70% and 100% of regrind into the virgin material. They concluded that yield stress and impact toughness reduced since the composite material become more fragile and prone to cracking. Spicker et al. [6] investigated the influence of multiple reprocessing cycles on the rheological, thermal and mechanical properties of PP manufacturing scrap. Melt flow rate (MFR), dynamic rheology, gel permeation chromatography (GPC), DSC, tensile tests, color analysis, and Fourier transform infrared spectroscopy (FTIR) were part of their experimental characterization technologies used to evaluate the material degradation properties. An increase of up to three times in the MFR was achieved with a 33.8% reduction of the mass average molecular weight. Wagner et al. [7] developed a case of study for post-consumer acrylonitrile butadiene styrene (ABS) mechanical recycling. They reground plastic housings of TVs and molded tensile specimens and thin wall boxes and then, performed tensile and impact tests and concluded that ABS could be reused since the material properties are better than those attain from commercial recycled resins. They also found some impurities and aesthetic defects. The effect of using antioxidants on two commercial ABS polymer types was investigated in [8]. They conducted a factorial analysis of variance (ANOVA) to evaluate the influence of the combination of different antioxidants, the polymerization method, and its interaction of the materials stability. They concluded that antioxidants added in sufficient amount during the original material processing reduce thermal-oxidative degradation improving the recycled material physical and chemical properties. In addition, Fioro et al. [9] studied ABS polymer degradation with different stabilization systems and repeatedly exposed the injected molded parts to ultraviolet A radiation, and mechanical recycling, in order to investigate the impact of these treatments on the physicochemical, thermo-oxidative, and mechanical properties. They found that mechanical recycling increases the injected molded parts strain at break with a slight decrease in the tensile modulus.

The results of previous research indicate a degradation of the mechanical properties, however, discoveries in materials science and manufacturing technologies have emerged. The pursuit of miniaturization of devices has brought the development of such processes that could cause less degradation allowing to work with superior polymeric blends by using emerging technologies, like the case of ultrasonic micro injection molding (UMIM). UMIM is a fast and low-cost new alternative process, that implements ultrasonic vibration to melt the resin and inject it into a mold [10]. This new manufacturing technology has several similarities with ultrasonic welding, which has been used as a starting point to identify the phenomena behind heating mechanisms, such as interfacial friction and volumetric heating [11]. Additionally, it is an analog process of the conventional IM, with a couple of relevant differences. In this case, instead of dosing and melting more material than needed, just the amount of material required for one shot is placed inside the chamber. The heating in the chamber is not external or by the motion of a screw; instead of that, an element called a sonotrode [12] vibrates uniaxial at ultrasound frequencies usually between 20 and 40 kHz inside the chamber, having its tip in contact with the plastic pellets and transmitting its mechanical oscillatory energy to the bulk pellets until these are melt. Thus, the molten polymer is injected directly into a cavity by the motion of a plunger. This technology transfers ultrasonic vibrational waves to the polymer pellets located in the machine chamber, and then this mechanical energy is converted cyclically into thermal energy that creates a polymer melt flow that replicates with accuracy the mold geometry during the mold filling and cooling phases. UMIM technology significantly reduces the energy required for the melting of the polymer since the machine components have no need to be preheated. Material degradation is avoided since the material does not remain in a heated chamber that could affect its properties because of the chamber temperature. Other advantages of this technology include the reduction of material wastage, risk reduction of cross contamination, and enhanced flowability of the melt polymer, and superior tensile strength mechanical properties. 

**Figure 1:**
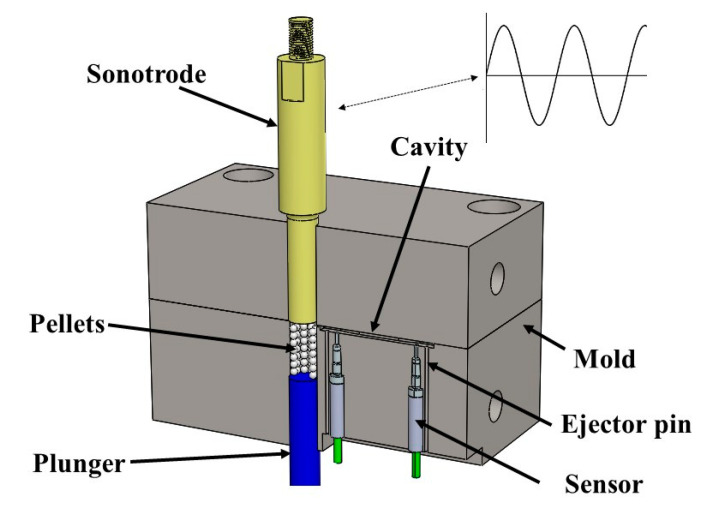
Ultrasonic micro injection molding (UMIM) section view scheme. The pelletized material is pushed upwards by the plunger as the sonotrode vibrates. As the material melts, it is injected into the cavity. After a specific cooling time, the mold is opened, and the ejector pins release the specimen.

Figure 1 shows a scheme of the process. Sanchez et al. [13] proved that UMIM is a processing technique that can be used to produce composite parts. In their investigation, they were able to produce ultra-high molecular weight polyethylene (UHMWPE)/graphite composites with enhanced mechanical properties and thermal stability. Planellas et al. [10] demonstrated by electron microscopy observations and XRD experimentation that ultrasound delivers uniform exfoliated structures with high oriented clay nanosheets in the direction of the polymer flow. Other studies provide information about the processing parameter values used to produce micro components by using different polymeric materials with this technology [14,15]. Zeng et al. [16] suggest a possible improvement of the tensile strength and elongation of PP powders molded by using ultrasound, while Sánchez-Sánchez et al. obtained higher tensile strength and Young’s modulus mechanical properties for material samples of UHMWPE produced by UMIM [17]. However, and to the best of the author’s knowledge, there are no publications that deal with the investigation of the material properties of recycle polymers use to manufacture components by UMIM. Therefore, the aim of this article focuses on investigating of how the mechanical, thermal and rheological properties of polypropylene (PP) recycle material change when ultrasonic micro injection molding (UMIM) technology is used to produce samples reprocessed for six consecutive cycles. Experimental characterization by differential scanning calorimetry (DSC), thermogravimetric analysis (TGA), Fourier transform infrared (FTIR) spectra, and rheology tests show that the PP material can be recycled up to five times before some signs of mechanical and physical properties degradation are observed. 

## 2. Materials and Methods 

### 2.1. Materials and Part Geometry

**Figure 2:**
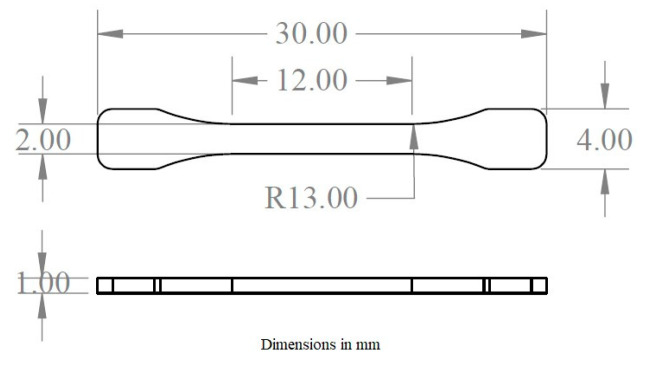
Scaled ASTM D638 tensile specimen.

Isotactic polypropylene (Axlene 12 homopolymer) for general injection purposes with a density of 0.9 g/cm^3^ and MFI of 12 g/10 min was purchased (Indelpro, Altamira, Mexico). The pellet particles have a semi-spherical shape with an approximate size of 5 mm. A dog-bone shaped microinjection mold cavity was fabricated to produce specimens for all the characterization experiments. The geometry was obtained from the ASTM D638 Standard for tensile properties of plastic specimens, adapted by a 1:5 scale reduction, displayed in Figure 2.

### 2.2. Molding Equipment

For this investigation, the ultrasonic microinjection molding machine Sonorus 1G, from Ultrasion (Barcelona, Spain) was used to produce the micro tensile specimens. The sonotrode frequency is fixed at 30 kHz, with a maximum amplitude of 56.2 µm.

### 2.3. Experimental Methodology

**Figure 3:**
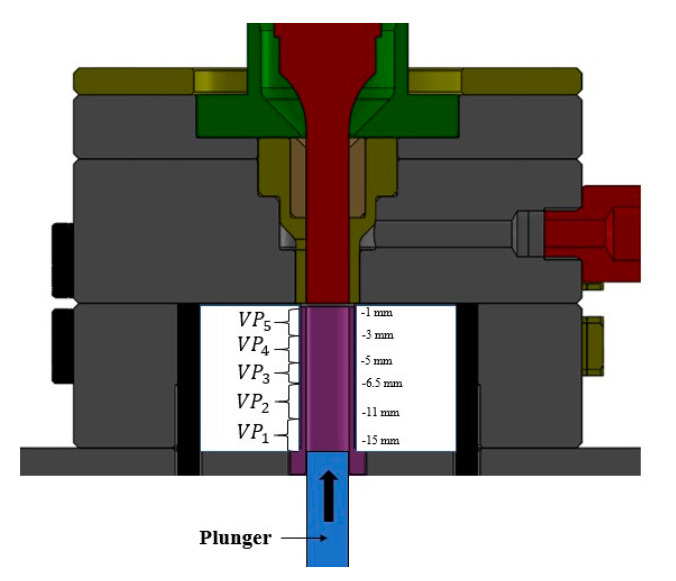
Plunger position and injection velocity profile. The distance from the plunger (in its retracted position) to the tip of the sonotrode is divided into five intervals that are used to control the injection velocity.

Experimental methodology is divided in four steps: (i) a screening phase; (ii) material grinding and preparation; (iii) full factorial design of experiments (DOE); and (iv) experimental characterization tests. In the screening phase, the main objective was to identify the technical parameters of interest and to set the values for producing full specimens that will be considered into factors and levels of a factorial design. The identified parameters were amplitude (A), injection (plunger) velocity profile (V), mold temperature (MT), and injection force (F). The corresponding values are displayed in Table 1. The injection velocity profile is an array of velocities whose values are executed for a user-defined interval of positions, as shown in Figure 3. For the amplitude parameter, consideration was made during the first instant on the plunger profile (−15 to −11 mm): for this, the sonotrode should remain off, in order to move the plunger upwards and reduce the free space between pellets before applying vibration to them; this decision is supported in the principles of the interfacial friction.

The screening parameters above were used to produce manufacturing specimens in the UMIM machine with shots of 0.36 g of virgin PP. Before molding, the virgin pellets were dried for 6 h in a Thermo Scientific Heratherm OGS180 oven at 80 °C and then left for 14 h in a desiccator. The criteria to determine if a combination was reliable was to produce five specimens for each combination. If three or more specimens are filled, the combination is marked as acceptable. Otherwise, it is discarded.

**Figure 4:**
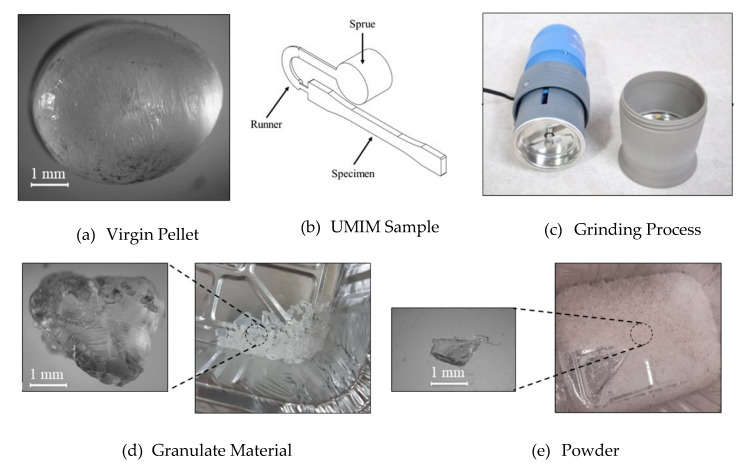
Material preparation process. (**a**) Virgin material; (**b**) produce specimens by UMIM; (**c**) IKA analytical mill; (**d**) granulate material; (**e**) powder morphology shape obtained after grinding polypropylene (PP) specimens.

An impact/attrition analytical mill (A11 basic) from IKA was used to grind the material. The material shape during the manufacturing process of the specimens was studied by considering the granulate and powder shape, as shown in Figure 4. 

After the regrind of the PP pellets, the measured granulate diameters were approximately between 1 mm < D_granulated_ ≤ 5 mm, and for the resulting powder material, we obtained a mean diameter value of D_powder_ ≤ 1 mm. The lasting milling time to obtain the desire PP shape did not exceed 8 s. 

A general full factorial design was performed based on the identified process parameter values of the screening stage to study the interactions and main factor effects, aiming to find an overall enhanced response, an analysis of variance (ANOVA) is applied. To investigate the mechanical and physical changes experienced by the recycle PP material after this was used to produce the specimens illustrated in Figure 4b via UMIM, five consecutive recycling stages (grinding–molding) were executed and evaluated.

### 2.4. Scanning Electron Microscopy (SEM)

Surface analysis was performed with a SEM microscope ZEISS EVO MA 25. The magnifications of 50×, 500× and 3000×, were used at 8 kV. The samples were sputtered with gold.

### 2.5. Mechanical Properties

To evaluate the tensile properties of the fabricated specimens was used an Instron 3365 universal testing machine with a load cell of 5 kN. The ASTM D638 standard was followed, in accordance with the specimen design. Tests speed of 10 mm/min was set. The experiments were carried out at room temperature (23 °C) and the material toughness was calculated by using the following equation

(1) $$\frac{Energy}{Volume}=\int_{0}^{\epsilon_{f}}\sigma d\epsilon$$

where *σ* is the engineering stress, *ε* is the unit strain, and $\varepsilon_{f}$ is the unit strain at the material fracture point [18].

### 2.6. Thermogravimetric Analysis (TGA)

Thermogravimetric analysis was performed using a PerkinElmer (Pyris 1) analyzer, considering a temperature interval between 30 to 500 °C at a heating rate of 10 °C/min. Nitrogen gas flow was set to 20 mL/min. Samples recorded weight was 5 mg.

### 2.7. Fourier Transformation Infrared Spectroscopy (FTIR)

FTIR analysis was performed using a PerkinElmer (Frontier) spectrometer equipped with a UATR accessory for surface analysis. The samples were placed over the ZnSe-diamond crystal of the UATR and scanned 32 times between 4000 and 400 cm^−1^, with a resolution of 4 cm^−1^. All measurements were performed by subtracting the baseline.

### 2.8. Differential Scanning Calorimetry (DSC)

DSC measurements were performed using a PerkinElmer (DSC 8000) instrument. The temperature program went from 30 to 200 °C at a heating rate of 10 °C/min, held at 200 °C for 3 min, and cooled down from 200 to 30 °C at 10 °C/min. The nitrogen gas flow rate was set to 20 mL/min. The specimens with 5 mg of weight were held in standard aluminum pans and covers. The degree of crystallinity, *X*_C_%, was calculated using the following equation

(2) $$X_{C}\%=100\left(\frac{\Delta H_{m}}{\Delta H_{m}^{0}}\right),$$

where ∆*H*_m_ is the melting enthalpy calculated from the area of the endotherm peak and ∆*H*_m_^0^ = 207 J/g is the enthalpy for a fully crystalline PP [19].

### 2.9. Rheological Properties

Dynamic rheology was performed using an Anton Paar Physica MCR 101 rheometer, equipped with an electrical heating device and a parallel plate geometry (PP25) of 25 mm diameter. The ultrasonically molded samples and pellets were stacked up over the bottom plate, molten, pressed with the upper plate and trimmed to achieve a 25 mm diameter disc specimen. The measuring gap between plates was set at 1 mm and the temperature to 200 °C. First, an amplitude sweep was executed for a 100% strain in order to find the linear viscoelastic range (LVE-R) of the samples. After this, an LVE-R of 5% strain was selected to perform frequency sweeps, varying the frequency logarithmically from 628 s^−1^ to 0.1 s^−1^ (100 to 0.0159 Hz) with 20 measuring points per decade. The results from the frequency sweeps were employed to calculate the molecular weight with the equation:

(3) $$\eta_{0}=k_{e}M_{w}^{a}$$

where *k*_e_ is a material constant (2.5 × 10^−17^ for isotactic PP at 200 °C), *M*_w_ is the weight average molecular weight, and *a* is the relaxation time exponent (3.6 for isotactic PP at 200 °C).

Since the molecular weight of PP influences the material reproducibility, a rheological method was developed by Zeichner and Patel [20] to determine its value by performing dynamic shear tests. Thus, the Polydispersity Index (PDI) was introduced to correlate the ratio of the molecular weight to the average molecular weight (*M*_n_) via the material crossover modulus by using the expression:

(4) $$PDI=\frac{100000}{G_{C}}$$

where *G*_c_ is the modulus measured in Pascals, at the crossover frequency where the storage modulus (*G*’) is equal to the loss modulus (*G*”). Experimental results validate the accuracy of the PDI value with *M*_w_/*M*_n_ for PP resins [21].

During experimental tests, the polymer specimen is deformed periodically with an angular frequency (ω) and thus, the material shear stress can be determined from:

(5) $$\sigma \left(t\right)=G^{*}\left(\omega \right)\gamma \left(t\right)=\eta^{*}\left(\omega \right)\dot{\gamma }\left(t\right)$$

Besides, the relationship between the complex viscosity and the material shear rate is determined from the Cox–Merz rule:

(6) $$\eta^{*}\left(\omega \right)=\eta^{*}\left(\dot{\gamma }\right)$$

that is applied to validate, using experimental data, the zero shear viscosity plateau and conjunction with the Wagner–Cauchy stress tensor constitutive model:

(7) $$\sigma \left(t\right)=-pI+\int_{-\infty }^{t}\mu \left(t-t^{\prime }\right)h\left(I_{1},I_{2}\right)B\left(t^{\prime }\right)dt^{\prime }$$

where *p* is an undetermined pressure, I is the identity tensor, *µ*(*t* − *t*^0^) is the memory function between any two times *t* and *t*^0^, and *h*(*I*_1_, *I*_2_) is the strain damping function that depends on the first and second invariants of the left Cauchy–Green deformation tensor for shear B(*t*’).

The plateau modulus ($G_{N}^{0}$) was computed by considering the following equation

(8) $$G_{N}^{0}=G_{FT}\left(0\right)-G_{e}=\frac{2}{\pi }\int_{-\infty }^{\infty }G_{FT}^{\prime \prime }\left(\omega \right)dln\left(\omega \right)$$

which is based on the use of the fluctuation-dissipation theorem, in order to obtain the material approximate entanglement molecular weight. Here, *GFT*(*t*) is the flow transition, which is the highest part of the relaxation function, *G*_e_ is the equilibrium modulus that is commonly set to zero. Then, the expression can be transformed into the integral of the loss modulus, *G*’’(*ω*), and the natural logarithm of the angular frequency, *ω*.

## 3. Results and Discussion

### 3.1. Design of Experiments

The combinations that passed the reliability test are summarized in Table 2. From the results, it is observed that the most repeated values were 90%, V_1_, 60 °C and 3000 N. By categorizing the values as high and low, a repeatable combination should exhibit a low injection speed, high mold temperature and low injection force. Based on the previous statements, and because at 80% amplitude it was most often not enough to melt the material, the amplitude of 90% was tested in a new stage with a new slightly reduced velocity profile V_4_: (2.5, 2.5, 2.5, 5.5, 5.5, 20) and 80 °C for the mold temperature. The injection force was maintained at 3000 N.

**Figure 5:**
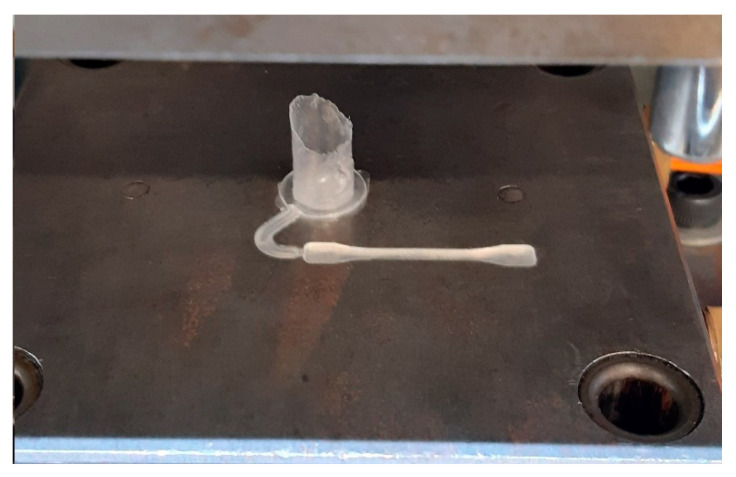
Complete specimen with homogenous and translucid color before ejection.

The new proposed combination 90/V_4_/80/3000 delivered much more repeatable results since the produced specimens are complete, homogeneous with translucent coloring, as shown in Figure 5. Therefore, it is concluded that the machine parameters of 90/V_4_/80/3000 provide the best combination to produce recyclable specimens for the DOE first stage.

The proposed DOE is a general full factorial design (Table 3), employing two levels for A, V and MT; and three levels for the specimen condition (virgin, granulate and powder). The injection force was fixed at 3000 N. The combinations of the DOE resulted in 24 base runs, which were randomly ordered using two replicates by using the Minitab software. Seven complete specimens (repeats) were produced for each of the 48 runs, and the average of the mass was taken as a first approach for the data analysis, however, in order to have more robust evidence of the performance of the specimens, three other response variables were extracted from mechanical testing: yield stress, ultimate stress and strain.

**Figure 6:**
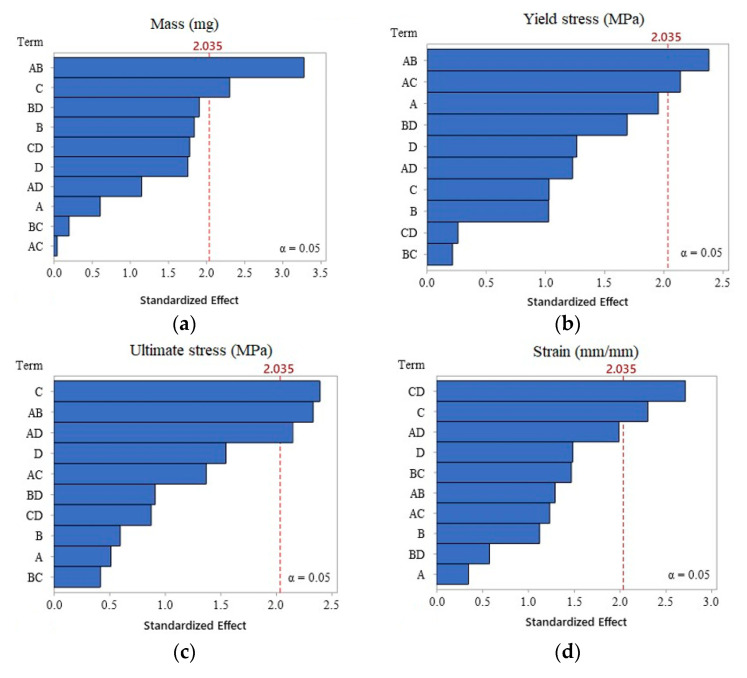
Pareto plots for specimen: (**a**) mass, (**b**) yield stress, (**c**) ultimate stress, and (**d**) strain. Here, A: amplitude; B: shape; C: velocity; D: mold temperature.

The results for all the experiments sequence are displayed in Table 4, the last four columns show the response variables of interest to proceed to the statistical study. An analysis of variance (ANOVA) was selected to determine the effects of the four process parameters over the different responses of the specimen. To support these part of the analysis, a Pareto chart shown in Figure 6 was generated in order to visualize the influence of the parameters and their interactions. It was confirmed that the injection velocity and the amplitude–shape interaction are the most recurrent parameters since they surpass the boundary effect of strain value of 2.035. The fact that the amplitude–shape interaction has a statistically significant effect confirms the importance of the initial pellet shape during the plasticizing stage for the UMIM manufacturing process in which the pellet shape influences the interfacial friction.

**Figure 7:**
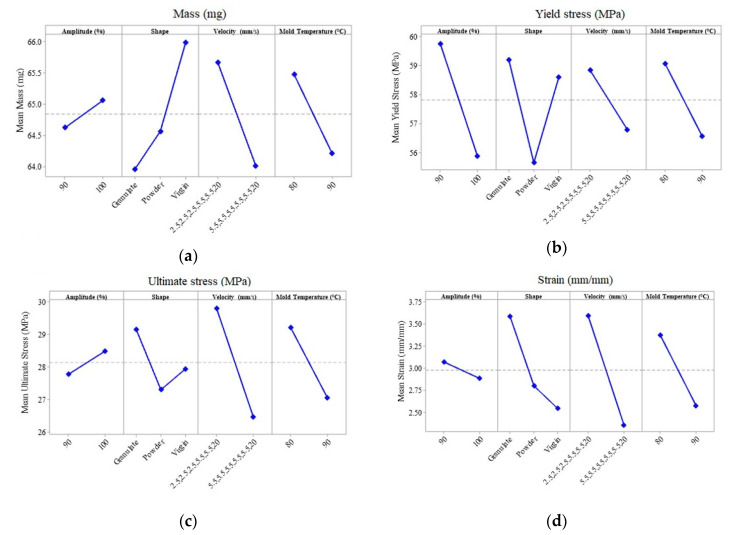
Main effects plot for the four selected response variables: (**a**) mass, (**b**) yield stress, (**c**) ultimate stress, and (**d**) strain. All the plots coincide in a [2.5, 2.5, 2.5, 5.5, 5.5, 20] injection velocity profile and a temperature of 80 °C to enhance the responses. The amplitude could statistically be either 90% or 100%. However, a complete evaluation is explored through microscopy and mechanical testing.

To achieve the manufacturing goals of the full factorial DOE executed, the main effects plot for each response is summarized in Figure 7. Notice from Figure 7 that for the machine parameter values set for the UMIM for the PP specimens, an enhancement of the mechanical properties is achieved. Furthermore, the samples produced with virgin material are more homogeneous since their mass tends to increase. These results agree with the selection of the best combination of machine parameter values set for any initial pellet shape, injection velocity profile, and mold temperature. Moreover, the oscillation amplitude, the specimen mass, and its ultimate stress value suggest that the 100% level of amplitude represents the best option, while the yield stress and strain suggest a maximum amplitude value of 90%. It can be concluded because of the Pareto chart results, that both amplitude value percentages are enough to produce a satisfactory specimen. This conclusion can be proved by running experimental characterization tests to confirm specimens’ enhancement of their physical and mechanical properties.

### 3.2. Microscopy Tests

**Figure 8:**
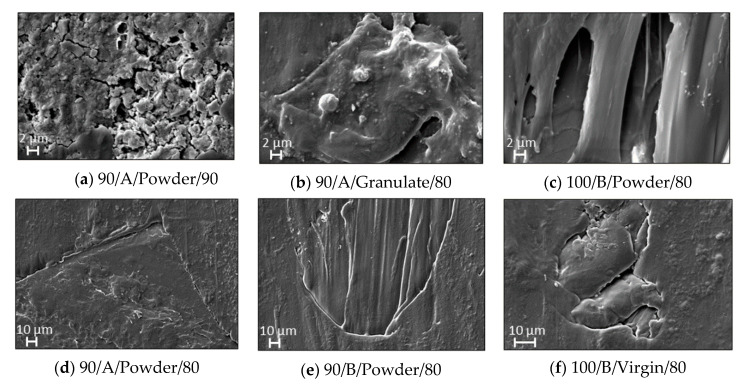
Worst defects found in samples from the base runs. (**a**–**c**) are porosity defects caused mainly by insufficient ultrasonic energy (due to amplitude). Images (**d**–**f**) show flow marks mainly due to high injection velocity.

**Figure 9:**
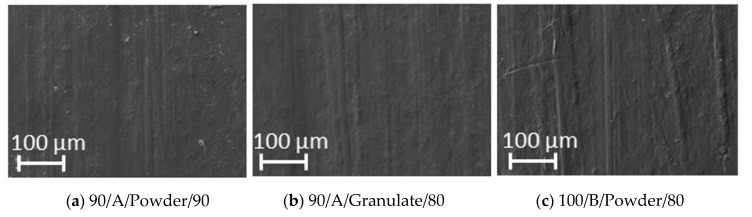
SEM micrographs comparison for the (**a**) virgin, (**b**) granulate and (**c**) powder materials. A homogeneous surface is observed without the presence of flow marks or porosities. No significant difference between the recycled samples and the virgin samples is observed. The micrographs suggest that a proper parameter combination in UMIM can produce homogeneous samples without surface finishing defects, even in the recycled materials.

SEM microscope experimental tests were performed in the produced specimens in order to find the most severe morphological defects, make a classification, and evaluate them. Two types of defects were found in some of the samples: porosity and weld lines/flow defects. Figure 8 shows the worst-case scenarios. Amplitude of 90% seem not to provide enough power for melting the full material. Notice that some specimen porosities appear since the material did not melt uniformly. Additionally, a high injection velocity seems to leave flow marks or weld lines where two streams of the material melt flow with different temperature gradients. These observations agree with the results obtained from the ANOVA where it was found that injection velocity and the interaction of amplitude-shape have significant effects in the samples. Even though no macroscopic cracks or bubbles were found inside the specimens, these micro defects appeared in the surface with dimensions between 1 and 100 µm. Figure 9 shows the surface image at a magnification of 100× in the center region of the specimens. No significant difference is found between the samples with 1-cycle regrind and the virgin one.

Additionally, none of the previously shown defects were found in any of the three groups of specimens. Based on these results, the combination (100/A/Shape/80) obtained through the Main-Effect plots is considered the best option to produce specimens with enhancing physical and mechanical properties. However, the specimens with microscopic defects will be subjected to mechanical tests to investigate if these micro defects affect the overall performance of the produced specimens by acting as stress concentration factors. 

### 3.3. Mechanical Properties

**Figure 10:**
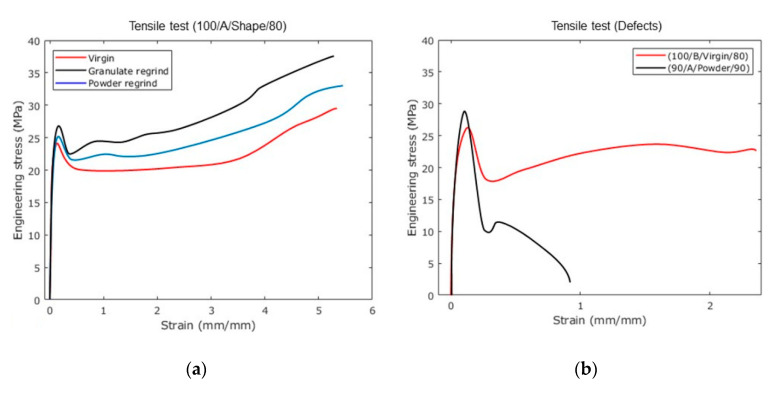
Stress–strain curves for virgin and recycled PP. (**a**) shows a significant increment of the tensile strength for the (1-cycle) recycled material. A slight improvement in strain is obtained for the powder samples. (**b**) Decreasing mechanical properties in specimens produced from PP powder by applying high injection velocity and low vibration amplitudes. The low strain value observed could be due to porous formation in the produced specimens that cause stress concentrations.

Mechanical testing of the samples produced in the base runs of the DOE were performed to find correlations between the characteristics observed with the SEM micrographs and the results of the ANOVA. Figure 10a shows the stress–strain average curves of 5 specimens tested for the best combination, according to the main effects plot and microscopy (100/A/Shape/80), for each set of materials. Surprisingly, the samples made with recycled material show enhance mechanical properties, as listed in Table 5. Notice that the recycled granulate material samples exhibit the highest tensile strength value when compare to the reference virgin material sample. In fact, its mechanical properties show an increase of 3.07% for Young’s modulus, 10.97% for yield stress, and 27.33% for ultimate stress, and a slight reduction of 1.29% for strain at break, when compare to those of the virgin material samples. The specimen made from PP powder show an enhancement of the yield and ultimate stresses, and an increase of the maximum strain value. The improvement of these mechanical properties in the recycled samples suggest that ultrasound energy produces mechano-chemical material changes that will be investigated by FTIR and DSC experimental characterization tests.

Figure 10b illustrates the stress–strain curves plotted by considering two types of produced specimens: specimens produced with low vibration amplitudes and specimens manufactured with high injection velocity. Other combinations were neglected due to the premature failure of at least three of the five specimens during the experimental tests. Notice from Figure 10b that when high injection velocity is used during the production of PP specimens by UMIM, a reduction of the ductility of the specimens which is mainly due to the surface porosities and weld lines observed in the SEM images of Figure 8, induce stress concentration that leads to the sample premature failure. Based on the experimental data shown in Figure 10a,b, it is concluded the granulate shape provides better mechanical performance when compared to those specimens produced with PP powder, therefore, granulated PP material will be used for consecutive reprocessing cycles under the UMIM machine parameters of (100/A/Shape/80).

**Figure 11:**
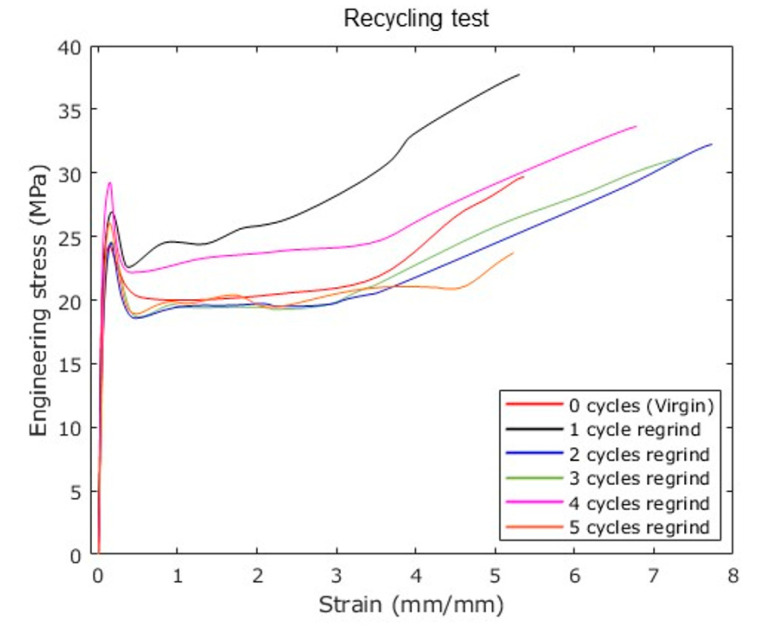
Stress–strain curves for five consecutive reprocessing cycles. Results shows that recycled material can withstand up to five cycles without showing a considerable reduction in mechanical properties.

Figure 11 shows the results of testing five cycles of granulated regrind material. In contrast to the specimens made with virgin PP (0 cycles regrind), the recycled samples exhibit superior stress–strain behavior, which agrees with the stress–strain plots shown in Figure 10a. Additionally, notice from Figure 11 that for samples made from granulated material regrind two or more cycles, the uniaxial stress decrease with an increase in the material ductility. Table 6 summarizes the results obtained for each reprocessing cycle. In Table 6, the toughness of the sample was calculated by integrating the area of the stress–strain curve, up to the strain at failure. This material property was calculated to provide a better comparison after recycling the material samples for 2 or more cycles. Based on the estimated toughness values, the produced recycle specimens are able to preserve their capacity to store deformation energy. However, the specimens manufactured with granulated PP and recycled five times exhibit a decrease in the ultimate stress and in the average toughness values, and show mechanical properties similar to those recorded from specimens made from virgin PP material.

### 3.4. TGA

**Figure 12:**
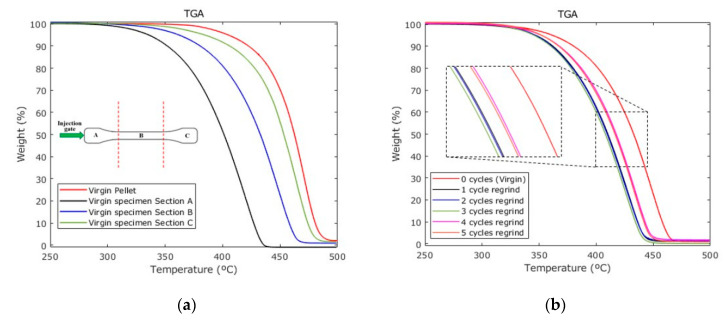
Thermal degradation of PP. (**a**) TGA measurements of three different sections of a specimen compared to the unprocessed material, the difference along the sample suggest an effect of the drop pressure and residence time. (**b**) TGA measurements performed in specimens manufactured by UMIM from recycle materials.

In order to evaluate how the thermal properties of the specimens vary along their length, each sample was divided into three sections, as shown in Figure 12 and then, TGA experimental measurements were performed in each section by considering specimens made from virgin or recycled PP material. The recorded data are shown in Figure 12a,b for PP pellets, and for specimens produced with UMIM by using virgin and recycle material. Notice from Figure 12 that the regrind material experience some degradation between 250 and 300 °C. These experimental TGA curves confirm that UMIM modifies the material molecular structure, which agrees with the findings of Refs. [9,10]. The specimen section C (the furthest from injection gate) developed properties very similar to those of the virgin pellets, while the material located close to section A, experience the highest molecular weight loss. The variation of molecular weight along the samples is mainly due by the pressure gradients produced during the manufacturing ultrasonic micro injection molding, since the pressure in section A, closer to the plunger, is higher than the pressure acting in section C. 

The material sample in section C is the first layer that is melted and injected, which implies the lowest residence time in the chamber during the UMIM process and then, the thermal load is less than that of sections A and B. 

Figure 12b shows the TGA curves recorded in the specimens’ section B. A shift of 25 °C in the TGA plots occurs after the material has been recycled. Surprisingly, the samples manufacture with recycle material for four and five cycles exhibit the best thermal resistance than those samples made from one, two and three regrinding cycles, which could be due to a molecular chain rearrangement. Table 7 shows the TGA values of interest and the comparison of the thermal behavior of the recycle samples against raw PP material.

### 3.5. FTIR

**Figure 13:**
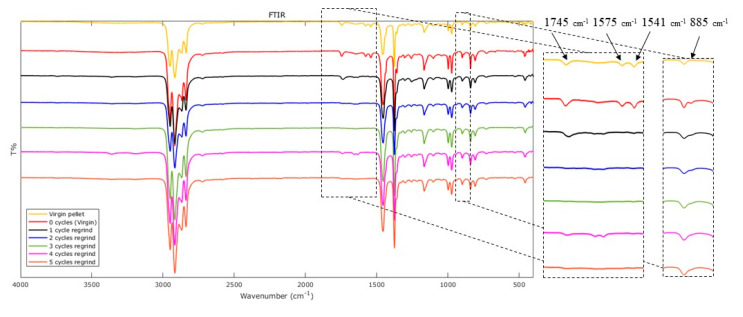
FTIR shows the comparison of the spectra between the reference material, the virgin specimen, and the recycled specimens.

The FTIR experimental curves collected from the reference material and the specimens produced by UMIM are shown in Figure 13. The peaks detected at 2915 and 1456 cm^−1^ correspond to the C–H stretching and C–H bending (methylene) groups, respectively, which are characteristic of PP. There is an increase of intensity in the 2915 cm^−1^ peak upon every reprocessing cycle, suggesting the presence of more C–H (alkane) groups. The results show that the ultrasonic oscillations do not shift the position of the infrared absorption peaks. However, the effect was evident in the molded specimens, mainly for the virgin material sample (0 cycle). After the first ultrasonic processing, the appearance of a peak in the region of 885 cm^−1^ occurs. This band is associated with a C=H bending from a vinylidene terminal group, which is originated by chain scission after thermal events. Interestingly, the peak vanishes after three molding cycles. As the material processing cycles increase, a disorder of molecular chains is likely to increase due to chains shortening. Additionally, thermomechanical effects are triggered, and the antioxidants react to prevent these reactions. Because of this, the intensity of the ester carbonyl band at 1745 cm^−1^, which is associated to the commonly used antioxidant for PP (Irganox 1010), was reduced due to the consecutive oxidative degradation, until it was mostly consumed after three processing cycles. The band at 1154 cm^−1^ is attributed to the amorphous phase of the isotactic PP. It can be observed a slight increase of intensity in this peak for the molded samples, indicating that the UMIM process made the PP more amorphous. The double band at 1575 and 1541 cm^−1^ is an indication of carboxylate group.

### 3.6. DSC

**Figure 14:**
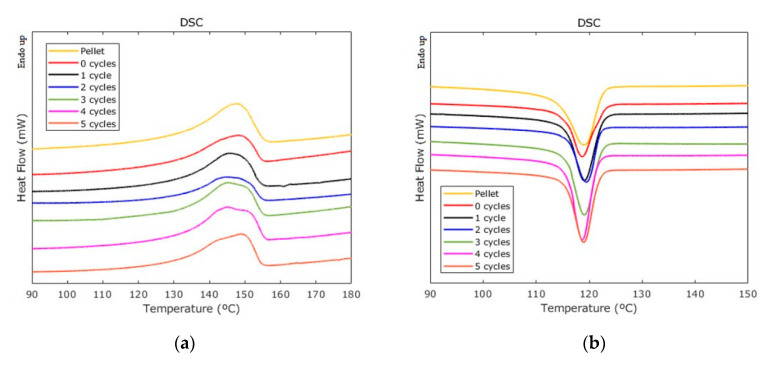
DSC experimental curves recorded for the reference material and for the recycle material: (**a**) melting temperature; (**b**) crystallization temperature.

DSC curves for the raw material and the UMIM samples are shown in Figure 14a for the melting temperature, and Figure 14b for the crystallization temperatures. With the enthalpy of the melting peak, the degrees of crystallinity were calculated from Equation (2). The corresponding values are listed in Table 8. These results suggest that ultrasonic vibration in combination with the injection velocity promotes the activity of molecular chains in such a way that the material entropy is increased when the raw material is processed. Notice that experimental data exhibit, for those regrind samples, an oscillating behavior of the crystallinity degree, perhaps associated with the variation of the molecular chains’ length over the recycling history and the perturbation of the ultrasonic waves. Nevertheless, the oscillating crystallinities for the processed samples are bounded in a narrow range between 30.4% and 33.8%. It is known that for a decrease of the crystallinity (more amorphous stage), the polymer becomes more stretchable due to the large entropy achieved during the regrind process. That it is confirmed with the experimental results observed in the specimens’ mechanical tests, shown in Figure 11, since the material samples made from two and three regrind cycles are the most amorphous samples and the ones with higher ductility.

### 3.7. Rheological Properties

**Figure 15:**
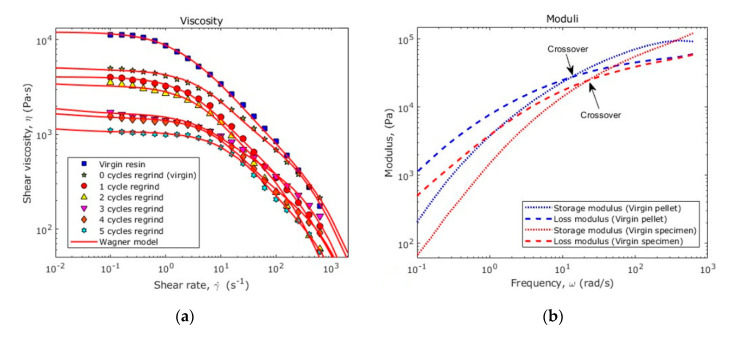
(**a**) Shear viscosity; (**b**) storage and loss moduli for the raw virgin PP and for the specimen produced with virgin PP material.

Figure 15a shows within markers the experimental shear viscosity obtained from the complex viscosity by the Cox–Merz rule. The solid red line represents the viscosity calculated, for each case, by using the Wagner model given by Equation (7). All the samples exhibit the typical shear thinning behavior. It is clear that after every cycle of material reprocessing, there is a reduction of the overall viscosity. These changes are a consequence of the variation of the molecular weight distribution and chain length in each sample, as listed in Table 9. 

**Figure 16:**
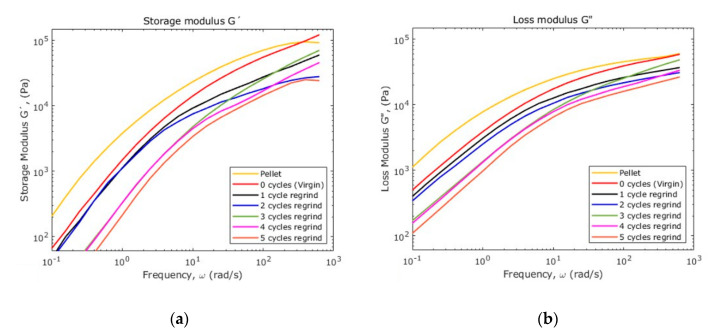
(**a**) Storage and (**b**) loss moduli for the consecutive recycling of PP.

The crossover modulus of recycled material recovers for the 2-recycles samples, demonstrating that, although the average chain-length was reduced, the ultrasonic energy modified the molecular order. Figure 16 shows how the storage and loss moduli vary along the material samples.

To visualize how the viscoelastic behavior changes over the shear rate, the loss tangent *δ* is used. This loss angle can be calculated from the equation

(9) $$\tan\delta =\frac{G^{\prime \prime }}{G^{\prime }}$$

**Figure 17:**
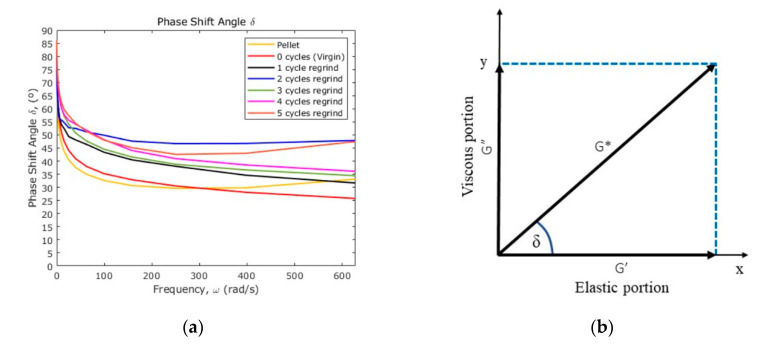
(**a**) Loss phase angle; (**b**) vector diagram for complex shear modulus.

Figure 17a shows the loss angle versus the frequency for all of the samples, while Figure 17b shows the vector representation of the complex modulus. When the value of *δ* is close to 0°, the material behavior is mostly elastic, but if the values of *δ* is close to 90° then, the material behavior is mostly viscous. From the curves shown in Figure 17a, the PP raw material exhibits the highest elastic behavior since this material possesses the highest percentage of crystallinity.

As the viscosity and surface tension of the polymer melt decrease upon recycling history, the propagation of the ultrasonic waves generates oscillatory pressure that induces a cavitation effect of bubbles expansion and contraction [22], causing a possible breaking of agglomerates [23] and lamellae, which could be the cause of the crystallinity reduction after the first ultrasonic material processing. The effect of ultrasound is also reflected in the polydispersity index value (PDI) that changes from 4.23 for the virgin sample to 5.58 due to the reduction of the chain length.

### 3.8. Molecular Entanglements

It is well-known that ultrasonic waves create a cavitation effect during ultrasonic injection molding [22,23]. In this sense, Pawlak et al. [23] found that there is a connection between cavitation (micro and nano cavities) and the polymer entanglement that modifies tensile properties. They also concluded that the degree of crystallinity is independent of the entanglement density. These findings confirm the strain hardening observed in Figure 11 for the different produced specimens because of the ultrasonic cavitation modifies the material entanglement density. Since both crystalline and amorphous components are involved, the interconnection of both phases determines the material mechanical properties [24]. 

The degree of entanglement can be measured by determining the average molecular mass between entanglement knots of chains in the polymer amorphous phase. Therefore, molecular measurements can be used to characterize strain hardening. In other words, a higher density of entanglements (less molecular weight between entanglements) results in a stronger strain hardening upon stretching of the molecular network, obviously, this is not the only factor that determines strain hardening, but it might help to understand this phenomenon.

**Figure 18:**
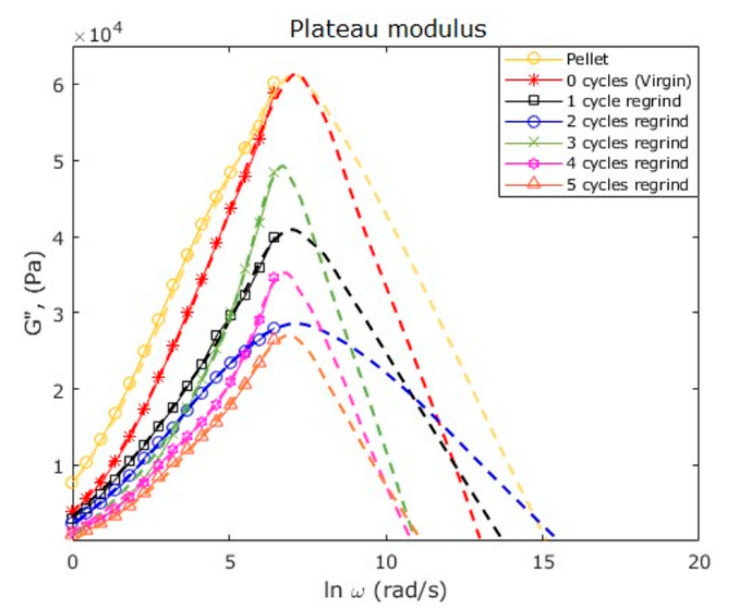
Plot of the loss modulus (*G*^’’^) versus the natural logarithm of frequency (ω), for the raw PP and the processing cycles, at 200 °C. The complete curves exhibit almost a symmetrical shape-form, satisfying the criteria needed to estimate the plateau modulus with Equation (10).

The plateau modulus, $G_{N}^{0}$, that describes the molecular architecture of polymers since its value depends on the material molecular weight between adjacent entanglements (*M*_e_), is defined as [20]:

(10) $$G_{N}^{0}=\frac{4}{\pi }\int_{-\infty }^{\omega_{max}}G^{\prime \prime }\left(\omega \right)d\;ln\left(\omega \right)$$

where *G*^″^(*ω*) is the loss modulus obtained from the frequency sweeps. This equation is obtained by the INT method, under the assumption that for polydisperse polymers, *G*^00^ plotted versus the natural logarithm of the frequency gives almost a symmetrical peak. Therefore, the function is integrated up to ω_max_, and the whole expression of Equation (8) is doubled, giving the constant 4/π. A linear extrapolation was performed to obtain the complete peak based on G″, in order to validate if Equation (10) was suitable for our collected data. Figure 18 shows the peaks for the raw PP and the recycled materials. Data left curves plotted with different symbols were obtained from the rheometer tests data, while the right dashed lines of each curve were plotted by extrapolation. Notice from Figure 18 that the curves are almost symmetrical, therefore, $G_{N}^{0}$ can be estimated from Equation (10). Thus, the molecular weight between entanglements (*M*_e_) can be obtained using the following expression:

(11) $$G_{N}^{0}=\frac{\rho RT}{M_{e}}$$

where *R* is the universal gas constant (8.314 J mol^−1^ K^−1^), *T* is the temperature, in degrees Kelvin, at which the loss modulus was measured and ρ≈0.765 g/cm^3^ is the polymer density estimated for the temperature interval of 200 °C≤T≤404 °C.

Table 10 shows the plateau moduli, as well as the molecular weights between entanglements calculated from Equation (11) for each sample. Notice from Table 10 that the value of *M*_e_ obtained for the raw PP is almost the same reported in the literature for isotactic PP, which is approximately 6900 g/mol [25,26,27]. From the data obtained, it is observed that *M*_e_ is mainly driven by the *M*_w_, in other words, as the molecular chains become shorter, they tend to entangle less. Additionally, note from Table 10 that the molecular weight between entanglements varies among the recycle samples in a nonlinear form because of the molecular chain random conformations in a stress-free state.

## 4. Summary

Using ultrasound as a heating mechanism allows the processing of fully recycled materials (100% regrind). This is due to the residence time that the material experiences inside during its plastic flow from the chamber to the mold part. In traditional injection manufacturing processes, the plastic material is molten inside a barrel and its remains in that state for some period until it reaches the output. In UMIM, only the required amount of material is placed inside the melting cylinder, which is molten by the ultrasound vibrations to inject the material into the mold cavity, making the residence times smaller than a couple of seconds. Of course, a good screening stage combined with a full factorial DOE is a practical approach to overcome the undesired morphological defects, such as internal porosities and specimen flow marks.

High injection velocity allows for obtaining a better filling of the specimens. However, in this work, strong evidence of weld lines was found for high velocities, which implies that the selection of parameters is never a trivial task and must be supported by more than one characterization technique. Previous research had reported low mold temperatures of around 30 °C for PP. In this work, the screening stage started with a low level of 40 °C, which is a close value to those reported in the literature however, for a mold temperature of 60 °C, specimens free of flaws are obtained. With the determination of the best combination of machine parameter values, it was possible to set the parameter values needed for the full factorial DOE. Through the Pareto charts, it is confirmed that the injection velocity and the amplitude–shape interaction are the recurrent parameters. The statistical significance of the amplitude–shape interaction help us to validate that ultrasonic injection is sensitive to the initial pellet shape. Since the particle size is small in PP powder material, the contact surface area among powder particles increase and therefore, less energy is required to heat the material. Of course, during grinding of recycle PP parts, small size particles could be obtained for increasing grinding times and thus, a careful selection of the particle size and grinding time should be done in order to obtain the best mechanical properties of the produced part by UMIM.

On the other hand, an amplitude of 90% seems not to generate enough power since some surface porosities were observed. In fact, high injection velocity seems to create weld lines where two streams of melt flow overlapped at different temperatures, or because the shearing rate of the polymer layers was very intense. The defects observed during experimental tests agree with the results obtained from the ANOVA analysis in which that injection velocity and the interaction of amplitude–shape have significant effects in the material samples. Then, if the low amplitude and high injection velocity are considered for the UMIM process, a reduction of the ductility of the sample is achieved, contrary to the machine parameter values set for the UMIM of the PP specimens by using the main effect plots that enhance the specimens mechanical properties by using the 100% level of amplitude. Specimens were produced by using recycled PP material that was regrinded for up to five cycles.

Uniaxial extension tests in the produced specimens indicate that the samples made with recycled material show better mechanical properties than virgin material. Experimental data confirm that the granulate shaped regrind material exhibit higher mechanical properties when compared to those collected from specimens produced from PP virgin material, with an increase of 3.07% for Young’s modulus, 10.97% for yield stress and 27.33% for ultimate stress, with a slight reduction of 1.29% for the elongation at break. The enhancement of mechanical properties of the recycled samples suggested possible mechano-chemical effects or changes in molecular crystallinity due to ultrasound, as discussed above.

## 5. Conclusions

In this study, it was investigated if UMIM is a suitable technology to produce pure and recycled PP micro components without compromising its physical and mechanical properties in PP materials that were recycled for up to five times. The combination of a screening phase with a full factorial design of experiments demonstrates that the surface and inner defects of molded specimens can be diminished if the evaluation of multiple response variables achieves an enhancing machine parameter combination. From the two regrind morphologies tested, the granulate showed better properties over the powder one, probably due to the surface roughness and irregularity of the shape. SEM images showed that improper molding parameters could produce micro defects that act as stress concentration factors that lead to early part failure.

Experimental results show that mechanical properties of the recycled granulated shape polypropylene specimens increased by 36%, 20%, 13%, 26%, and 48% in Young Modulus, yield stress, ultimate stress values, deformation strain, and toughness, respectively. 

FTIR analysis revealed that oxidative degradation occurred upon material reprocessing by observing in the corresponding spectra, the absence of the peak (1745 cm^−1^), which is associated to the antioxidant additives (Irganox 1010) used in the Ziegler–Natta polymerization of PP resins. The DSC analysis showed that the degree of crystallinity of the raw PP was reduced by the ultrasound, making the material more amorphous. Upon recycling, the percentage of material crystallinity oscillates between 30.4% and 33.8%, probably due to cavitation effects. From TGA measurements, it was observed that the thermal stability of UMIM recycled samples was not significantly influenced, while from rheology tests a decrease of the molecular weight was found, caused by chain scission and, consequently, an overall reduction of the shear viscosity. 

After 5 reprocessing cycles (6 UMIM cycles in total) the molecular weight of the PP was reduced in 48%, with the most abrupt decrease during the transformation of virgin PP. Additionally, it was observed that the average molecular weight drives the molecular weight between entanglements and thus, as the molecular chains became shorter, the tendency of entanglements per chain length becomes smaller. In fact, we found that samples’ molecular weight varied with the number of recycling cycles in nonlinear form. The highest reduction in molecular weight occurs when the specimens are fabricated from raw PP material. In this case, viscosity curves show after every remolding, a typical shear-thinning behavior, with an overall reduction of the material viscosity. We also found that recycled specimens crossover moduli varied along with the recycling history, demonstrating that, although the average length size of molecular chains is reduced, the ultrasound energy modified the molecular order. This effect is verified by the variation of the PDI values because of the shortening and homogenization of the material chains.

Therefore, it can be concluded that the UMIM parameter values are linked to the molecular weight, molecular entanglements, and the degree of crystallinity of recycled samples, which modify their mechanical properties.

## Figures and Tables

**Table 1:** Screening table used to test combinations and obtain proper values for the design of experiment (DOE). The injection velocity profiles are V1: [2.5, 2.5, 5.5, 5.5, 5.5, 20], V2: [5.5, 5.5, 5.5, 5.5, 5.5, 20] and V3: [6, 6, 6, 7, 8, 20] (mm/s).

Parameter	Experiments		
A (%)	80	90	100
V (mm/s)	V_1_, V_2_, V_3_	V_1_, V_2_, V_3_	V_1_, V_2_, V_3_
MT (°C)	40, 60	40, 60	40, 60
F (N)	3000, 6000	3000, 6000	3000, 6000

**Table 2:** Screening combinations that delivered at least three complete specimens, denoted by a letter C.

Combination	1	2	3	4	5
80/V_2_/40/3000	C	C	C	-	-
90/V_1_/60/6000	-	C	-	C	C
90/V_3_/60/3000	C	-	C	-	C
100/V_1_/60/3000	-	-	C	C	C

**Table 3:** General full factorial design. The injection velocity profiles are V_A_: [2.5, 2.5, 2.5, 5.5, 5.5, 20], V_B_: [5.5, 5.5, 5.5, 5.5, 5.5, 20].

Parameter	Level 1	Level 2	Level 3
A (%)	90	100	-
V (mm/s)	A	B	-
MT (°C)	80	90	-
F (N)	3000	3000	3000
Shape	Virgin	Granulate	Powder

**Table 4:** General full factorial design. The injection velocity profiles are V_A_: [2.5, 2.5, 2.5, 5.5, 5.5, 20], V_B_: [5.5, 5.5, 5.5, 5.5, 5.5, 20].

Label	A (%)	Shape	V (mm/s)	MT (°C)	Mass (mg)	Yield Stress (MPa)	Strain (mm/mm)	Ultimate Stress (MPa)
R1	90	Virgin	A	90	68.461	84.577	8.024	40.276
R2	90	Virgin	B	80	72.561	66.831	1.389	26.367
R3	100	Granulate	A	80	67.284	59.462	4.025	29.501
R4	90	Granulate	B	90	56.396	53.879	2.361	26.950
R5	100	Granulate	B	80	62.539	62.211	4.017	35.205
R6	90	Powder	A	90	64.207	55.117	0.606	23.241
R7	90	Granulate	B	80	66.167	54.101	4.152	29.653
R8	100	Powder	A	80	65.783	59.266	6.775	38.073
R9	100	Virgin	A	90	64.986	46.447	0.085	21.398
R10	100	Virgin	B	90	63.267	45.128	0.067	23.551
R11	100	Powder	B	90	65.299	52.442	2.655	23.252
R12	100	Powder	A	90	66.556	57.060	2.788	27.916
R13	90	Powder	B	80	62.777	51.780	2.551	24.314
R14	90	Virgin	B	90	61.440	56.993	3.383	28.283
R15	100	Granulate	B	90	64.097	57.665	6.829	28.211
R16	90	Granulate	A	80	64.357	54.444	2.704	23.658
R17	90	Granulate	A	90	63.414	64.913	3.038	32.218
R18	100	Virgin	B	80	64.204	69.030	2.966	34.959
R19	100	Virgin	A	80	64.499	57.884	6.125	33.014
R20	90	Powder	B	90	65.531	60.055	6.024	22.758
R21	90	Virgin	A	80	67.886	56.789	5.505	32.844
R22	90	Powder	A	80	66.747	64.658	6.345	31.619
R23	100	Granulate	A	90	66.311	62.364	3.078	31.551
R24	100	Powder	B	80	63.964	49.737	0.188	25.641
R1	90	Virgin	A	90	67.379	58.931	4.186	27.080
R2	90	Virgin	B	80	71.747	61.247	0.263	24.870
R3	100	Granulate	A	80	68.024	62.255	4.475	40.750
R4	90	Granulate	B	90	57.921	59.702	2.237	28.448
R5	100	Granulate	B	80	64.630	63.248	4.567	31.328
R6	90	Powder	A	90	64.611	54.987	0.300	27.031
R7	90	Granulate	B	80	66.967	57.388	0.173	14.223
R8	100	Powder	A	80	68.073	54.888	4.900	31.527
R9	100	Virgin	A	90	66.756	41.274	0.057	24.320
R10	100	Virgin	B	90	62.267	54.971	1.223	22.516
R11	100	Powder	B	90	65.494	56.860	1.184	25.397
R12	100	Powder	A	90	66.723	49.939	0.203	26.039
R13	90	Powder	B	80	57.559	55.175	0.163	25.456
R14	90	Virgin	B	90	63.689	46.060	0.115	20.442
R15	100	Granulate	B	90	64.533	63.504	3.530	32.127
R16	90	Granulate	A	80	62.027	53.452	4.940	26.374
R17	90	Granulate	A	90	66.897	65.330	5.541	31.732
R18	100	Virgin	B	80	64.396	54.927	0.072	23.332
R19	100	Virgin	A	80	64.020	53.315	3.825	22.194
R20	90	Powder	B	90	63.003	56.085	2.642	29.942
R21	90	Virgin	A	80	68.390	83.062	3.404	41.963
R22	90	Powder	A	80	60.870	58.557	3.634	26.955
R23	100	Granulate	A	90	61.879	53.147	1.708	24.597
R24	100	Powder	B	80	65.954	53.990	3.856	27.842

**Table 5:** Experimental data confirm an improvement of the mechanical properties of recycled samples. The improvement percentage (in parenthesis) is determined with respect to the virgin PP material specimen properties.

Sample	Average Young’s Modulus (MPa)	Average Yield Stress (MPa)	Average Ultimate Stress (MPa)	Average Maximum Strain (mm/mm)
Virgin	561.39	24.14	29.67	5.348
Granulate	578.66 (+3.07%)	26.79 (+10.97%)	37.78 (+27.33%)	5.279 (−1.29%)
Powder	510.07 (−9.14%)	25.13 (+4.10%)	33.07 (+11.46%)	5.478 (+2.43%)

**Table 6:** The results show a strengthening of the recycled samples for 4-cycles regrind. Notice a slight reduction of the ultimate stress and average toughness values for samples made from material regrind 5-cycles.

Sample	Average Young’s Modulus (MPa)	Average Yield Stress (MPa)	Average Ultimate Stress (MPa)	Average Maximum Strain (mm/mm)	Average Toughness (MJ/m^3^)
0 cycles (virgin)	561.39	24.14	29.67	5.35	120.40
1 cycle regrind	578.66 (+3.07%)	26.79 (+10.97%)	37.78 (+27.33%)	5.28 (−1.30%)	151.33 (+25.68%)
2 cycles regrind	547.45 (−2.48%)	24.65 (+2.11%)	32.4 (+9.20%)	7.74 (+44.67%)	180.82 (+50.18%)
3 cycles regrind	517.15 (−7.88%)	24.47 (+1.36%)	31.23 (+5.25%)	7.31 (+36.63%)	171.03 (+42.05%)
4 cycles regrind	767.27 (+36.66%)	29.01 (+20.17%)	33.68 (+13.51%)	6.76 (+26.35%)	178.31 (+48.09%)
5 cycles regrind	679.79 (+21.09%)	25.98 (+7.62%)	23.67 (−20.22%)	5.23 (−2.24%)	107.24 (−10.93%)

**Table 7:** Thermogravimetric analysis performed in the raw reference material and in recycle material. There is a significant decrease of the temperature at which degradation starts. After the first recycling of the specimen made from raw PP material, the regrind material preserves its thermal resistance.

Sample	Degradation (°C)	50 wt % Loss (°C)	100 wt % Loss (°C)
0 cycles (virgin)	364	463	496
1 cycle regrind	280	433	477
2 cycle regrind	265	411	460
3 cycle regrind	250	410	460
4 cycle regrind	275	408	459
4 cycle regrind	285	417	465

**Table 8:** Results were obtained by differential scanning calorimetry (DSC) experimental characterization tests. The melting and crystallization temperatures show an oscillating behavior over the recycling history. The ultrasonic plasticizing made the material more amorphous, as shown in the degree of crystallinity achieved.

Sample	Melting Temperature (°C)	Enthalpy (J/g)	Crystallization Temperature (°C)	Crystallinity (%)
Virgin PP	148.0	75	118.7	36.2
0 cycles (virgin)	148.8	66	118.6	31.9
1 cycle regrind	146.0	70	119.1	33.8
2 cycles regrind	145.1	63	119.5	30.4
3 cycles regrind	145.0	65	119.1	31.4
4 cycles regrind	144.9	68	118.7	32.8
5 cycles regrind	148.9	67	119.0	32.3

**Table 9:** Molecular weight data obtained from experimental rheological tests.

Sample	*η*0 (Pa·s)	*M_w_* (g/mol)	Crossover Modulus (Pa)	PDI	*M_n_* (g/mol)
Virgin PP	12,040	556,148	27,000	3.70	150,310
0 cycles (virgin)	5059	437,109	23,600	4.23	103,335
1 cycle regrind	4057	411,114	24,300	4.11	100,027
2 cycles regrind	3417	391,969	25,000	4.00	97,992
3 cycles regrind	1873	331,682	23,300	4.29	77,315
4 cycles regrind	1652	320,313	19,400	5.15	62,197
5 cycles regrind	1144	289,232	17,900	5.58	51,834

**Table 10:** Molecular data regarding to plateau moduli, molecular weight between entanglements, weight average molecular weight, and polydispersity.

Sample	$G_{N}^{O}(\mathrm{Pa})$	*M_e_* (g/mol)	*M_w_* (g/mol)	*M_n_/M_n_*
Virgin PP(Pellets)	442,560	6798	556,148	3.70
0 cycles (virgin)	359,000	8380	437,109	4.23
1 cycle regrind	249,900	12,038	411,114	4.11
2 cycles regrind	199,840	15,054	391,969	4.00
3 cycles regrind	228,910	13,142	331,682	4.29
4 cycles regrind	172,810	17,409	320,313	5.15
5 cycles regrind	143,890	20,907	289,232	5.58

## Abbreviations

The following abbreviations are used in this manuscript.

UMIM	Ultrasonic Micro Injection Molding
IM	Injection Molding
PP	Polypropylene
DOE	Design of Experiments
PDI	Polydispersity Index
*M*_w_	Molecular Weight
*M*_e_	Molecular weight between entanglements
